# Quantitative metrics commonly derived from diffusion tractography covary with streamline length: a characterization and method of adjustment

**DOI:** 10.1007/s00429-024-02854-9

**Published:** 2024-09-11

**Authors:** Richard G. Carson, Alexander Leemans

**Affiliations:** 1 Trinity College Institute of Neuroscience and School of Psychology, Trinity College Dublin, Dublin 2 Dublin, Ireland; 2 Image Sciences Institute, University Medical Center Utrecht, Utrecht, 85500 The Netherlands

**Keywords:** Brain mapping, Data interpretation, Human, White matter, Neural pathways, Neuroanatomical tract-tracing techniques

## Abstract

Tractography algorithms are used extensively to delineate white matter structures, by operating on the voxel-wise information generated through the application of diffusion tensor imaging (DTI) or other models to diffusion weighted (DW) magnetic resonance imaging (MRI) data. Through statistical modelling, we demonstrate that these methods commonly yield substantial and systematic associations between streamline length and several tractography derived quantitative metrics, such as fractional anisotropy (FA). These associations may be described as piecewise linear. For streamlines shorter than an inflection point (determined for a group of tracts delineated for each individual brain), estimates of FA exhibit a positive linear relation with streamline length. For streamlines longer than the point of inflection, the association is weaker, with the slope of the relationship between streamline length and FA differing only marginally from zero. As the association is most pronounced for a range of streamline lengths encountered typically in DW imaging of the human brain (less than ~ 100 mm), our results suggest that some quantitative metrics derived from diffusion tractography have the potential to mislead, if variations in streamline length are not considered. A method is described, whereby an Akaike information weighted average of linear, Blackman and piecewise linear model predictions, may be used to compensate effectively for the association of FA (and other quantitative metrics) with streamline length, across the entire range of streamline lengths present in each specimen.

**Supplementary Information:**

The online version contains supplementary material available at 10.1007/s00429-024-02854-9.

## Introduction

Tractography algorithms are used extensively to delineate white matter structures, by operating on the voxel-wise information generated through the application of diffusion tensor imaging (DTI) or other models to diffusion weighted (DW) magnetic resonance imaging (MRI) data. With the aim of delineating only those tracks (consisting of “streamlines”) that are anatomically plausible, tractography algorithms incorporate certain criteria. For example, a curvature threshold (e.g., between 30° and 70°) may be applied to exclude deviations that would otherwise result in propagation to an adjacent streamline. Magnitude thresholding on fractional anisotropy (FA) values (or on the fibre orientation distribution (FOD) in the case of the constrained spherical deconvolution (CSD) model) results in the termination of tracking when the value for a voxel falls below the defined threshold (e.g., FA < 0.20).

In the present paper, we demonstrate that these methods yield substantial and systematic associations between streamline length and several tractography derived quantitative metrics, such as FA. These effects arise as FA or FOD values for successive voxels defining a streamline do not exhibit an abrupt transition from values well in excess of the magnitude threshold, to a value falling below the threshold. Rather, the distribution of values obtained for such measures tends to be relatively smooth, with the largest values being obtained close to the middle of the streamline, and the lowest values (by definition) at the two ends of the streamline (Zhang et al. 2018). It follows that the relative contribution of “low-values” to the total estimate will be larger for shorter streamlines (and fibre tracks) than for longer streamlines. We show that these factors give rise to an unambiguous and consistent relationship between the estimated FA values and streamline length.

It is not our purpose to engage in detailed biophysical modelling of this previously unreported phenomenon. Rather, as realised through statistical modelling, our objectives are to (1) demonstrate that the effect is clear and in practical terms far from trivial, and (2) outline the relatively straightforward steps that may be taken to compensate for its impact on quantitative metrics commonly derived from tractography.

## Methods

With a view to illustrating the key features of this phenomenon, we first present a re-analysis of data acquired in work presented by Ruddy et al. (2017). Analyses of additional data are described in the Supplementary Information. The relevant features of the data acquisition and pre-processing undertaken by Ruddy et al. (2017) are as follows.

The participants were forty-three neurologically healthy right-handed volunteers (aged 22.5 ± 2.9 SD, 28 female). All gave informed consent to procedures that (with the exception of preregistration) were in accordance with the Declaration of Helsinki. These had been approved by the appropriate Queen’s University Belfast and Trinity College Dublin Ethics Committees.

A 3 T Philips Achieva magnetic resonance scanner, with an eight-channel head coil, was used to acquire diffusion weighted images. The sequence comprised single shot echo planar imaging (EPI) with a slice thickness of 2.29 mm, repetition time = 9994 ms, echo time = 73 ms, number of diffusion directions = 61, b value = 1500 s/mm^2^, number of slices = 60 (transverse), in-plane resolution 2.3 × 2.3 mm^2^, with a field of view of 258 mm (RL) × 258 mm (AP) × 138 mm (FH).

ExploreDTI (Leemans et al. 2009) was used for data processing. Images were corrected for head movement and eddy currents using the procedure described in Leemans and Jones (2009). Tensor estimation was performed using the iteratively reweighted linear least squares approach (Veraart et al. 2013). Fibre trajectories were computed with CSD based tractography (Tournier et al. 2007). When compared to conventionally applied DTI based fiber tractography (FT), this method increases the sensitivity with which functionally significant variations in white matter characteristics may be detected (Reijmer et al. 2012). Recursive calibration of the response function was used to optimise the estimation of the fibre orientation distribution (FOD) functions (Tax et al. 2014). A uniform grid of tractography seed points at a resolution of 2 × 2 × 2 mm^3^ was employed, with an angle threshold of 30 degrees, an FOD threshold of 0.1, and a maximum harmonic order of eight.

The cortical motor network atlas developed in Ruddy et al. (2017) was used, including the following regions: posterior and anterior primary motor cortex (M1a and M1p), dorsal and ventral premotor cortex (PMd and PMv), supplementary motor area proper (SMA proper) and pre-supplementary motor area (pre-SMA), primary sensory cortex (S1), and the cingulate motor area (CMA) in both hemispheres. Reconstructed fibre trajectories for all pairwise combinations of brain regions were quantified separately for each individual participant. As a result, a total of 120 tracts were obtained: 64 transcallosal tracts, and 28 tracts within each hemisphere. We herein focus on the relationship between FA and streamline length. Analyses of other quantitative metrics frequently derived from diffusion tractography—apparent fibre density (AFD), radial diffusivity (RD) and mean diffusivity (MD), are described in the Supplementary Information.

## Results

As it is known that distributions of FA values (and indeed all eigenvalue-based measures (Babamoradi et al. 2013)) deviate from normality (Cascio et al. 2013; Clement-Spychala et al. 2010), robust statistical methods were used throughout. All reported confidence intervals (c.i.) are bias corrected adjusted (bca), based on 1000 bootstrap samples. In respect of correlations, these were converted to z scores before bootstrap resampling (Gorsuch and Lehmann 2010), the bca statistics were weighted by sample size (Karyawati et al. 2020), and the inverse transform then applied.

As a first step we calculated the Kendall rank correlation coefficient, to characterise the ordinal association of FA and streamline length. The magnitude of this coefficient expresses the similarity of the orderings of the two samples. This was done in two ways. In the first, the coefficient was calculated separately for each of the 43 participants. As streamlines were not resolved for all of the 120 tracts, the mean number of tract observations included in each calculation was 82 (95% c.i. 80–83). The mean value of tau – the Kendall correlation coefficient, was then obtained across participants. The mean magnitude of the correlation was 0.35 (95% c.i. 0.30–0.39), corresponding to an effect of “moderate” size. In other words, within individual brains, tracts with longer streamlines are characterised by moderately larger FA values. In the second method of analysis, the coefficient was calculated separately for each of the tracts (e.g., left M1a to right M1a), using the sample of 43 participants. The mean value of tau across all tracts was then derived. In this case, each bootstrapping sample comprised a random selection of tracts (rather than participants). The mean magnitude of the correlation was 0.28 (95% c.i. 0.24–0.31). Thus, when any given tract is considered across different brains, individuals with longer streamlines tend to exhibit larger FA values.

**Fig. 1:**
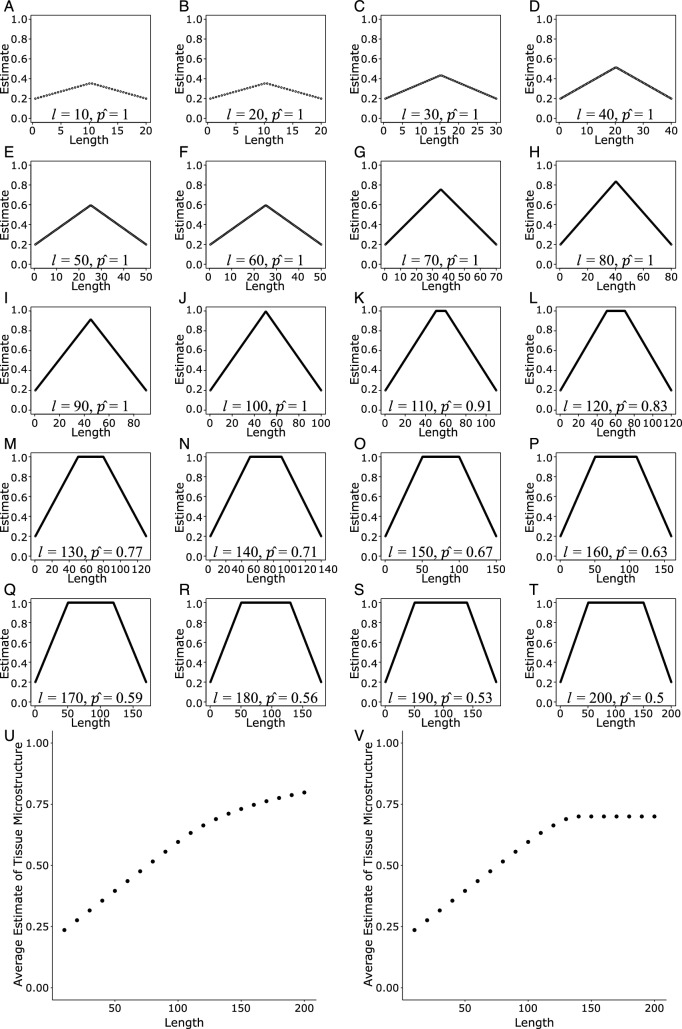
For illustrative purposes, the theoretical estimate of a quantitative metric, such as FA, along a streamline is modelled as increasing with streamline length from a termination magnitude threshold of 0.2, at a rate equivalent to an increase of 1.6 for every change of 100 units of length. It is further assumed that the quantitative metric is constrained to a range of 0 to 1. Given these parameters, the terminal sections (less than 50 units of length) – at both ends of the streamline, are characterised by all values being lower than 1. It is specified that in these regions the slope is constant, and independent of the overall length of the streamline. Within centre (non-terminal) sections, all values are equal to 1 (the theoretical asymptote). Average estimates of the quantitative metric of (i.e., for an entire streamline) will therefore increase in a linear fashion with increases in streamline length (Panels A to J), up to the length at which the upper limit of 1 is reached (i.e., encompassing only the two terminal sections). For streamlines in excess of this length (i.e., also encompassing a centre section) (Panels K to T), the estimate of the quantitative metric will then increase as a power function with further increments in greater streamline length (Panel U). Crucially however, the tissue of real specimens will be characterised by estimates of the quantitative metric (e.g., FA) that are lower than the theoretical average. That is, the values obtained empirically will not continue to increase towards the theoretical limit (1) with increases in streamline length. In the present illustration, a maximum value of 0.7 is assumed. It follows that for this specimen the empirical average will remain 0.7 for all streamline lengths for which the theoretical model predicts a value of 0.7 or above. It would be anticipated therefore that the average estimate of the quantitative metric will increase in a linear fashion until the streamline length at which a value of 0.7 is obtained and remain constant (slope equal to zero) for streamlines in excess of this length (Panel V). For Panels A to T, the length (*l*) of the streamline and the proportion (*p̂*) of the streamline comprised of terminal sections (less than 50 units of length) are indicated

It should not however be assumed that the magnitude of the association between FA (or AFD etc.) and streamline length remains constant across all streamline lengths. A simple model derived from basic assumptions illustrates this point (Fig. 1). We emphasise that this is a statistical, rather than a biophysical, model. It encompasses the reality of magnitude thresholding, whereby tracking is terminated when the value obtained for a voxel falls below a pre-defined threshold. It also includes the empirical observation that estimates of FA diminish progressively towards the ends of a streamline (Zhang et al. 2018), It can further be assumed that these estimates are constrained to a finite range (e.g., 0–1). Given this set of assumptions, it is predicted that average (or median) estimates of some idealised quantitative metric (i.e., for an entire streamline) will increase in a linear fashion with extensions of streamline length (Fig. 1, Panels A to J), up to the length at which the upper limit of the range of possible values (e.g., 1) is reached. For streamlines that exceed this length (Fig. 1, Panels K to T), the average estimate of the metric (for an entire streamline) will increase as a power function with further increments in streamline length (Fig. 1, Panel U). But a key caveat applies. Real biological specimens do not yield values with a magnitude equal to the upper limit of the potential range. Rather, for each specimen (i.e., for each individual brain) there will exist an asymptotic value for the derived estimate (Clark et al. 2001) (Fig. 1, Panel V). At streamline lengths beyond that at which the asymptote is obtained, no further increase in the average estimate of the quantitative metric (FA, AFD etc.), i.e., no dependency on streamline length, is to be expected.

**Fig. 2:**
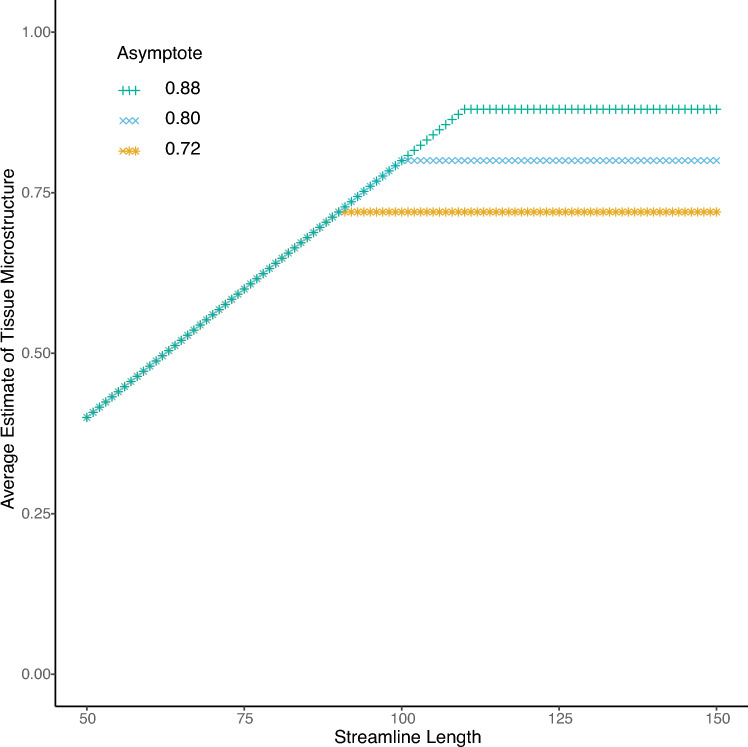
Theoretical fits to the Blackman (also known as a linear-plateau) function are illustrated. In this example, notional data from three specimens are shown to differ only with respect to the asymptotic value of the derived estimate of a quantitative metric (e.g., FA). Specimen 1 (orange *) exhibits an asymptotic value of 0.72; Specimen 2 (blue ×) exhibits an asymptotic value of 0.80; and Specimen 3 (green +) exhibits an asymptotic value of 0.88. The streamline length corresponding to the start of the plateau region (the point of inflection) varies accordingly. In these examples, the slope of the initial segment is equivalent in each case. Empirical fits derived using the Blackman function can however also vary with respect to the slope of the initial segment. Furthermore, variations in the asymptotic value can covary with the streamline length at which the start of the plateau region occurs (Archontoulis and Miguez 2015)

In line with this characterisation, non-linear curve fitting using the Blackman (also known as a linear-plateau) function (Archontoulis and Miguez 2015) (Fig. 2) to characterise the relationship between FA and streamline length indicated that in 41 of the 43 participants, the fit to the Blackman function was superior to that achieved using a linear model. It is however an intrinsic feature of the Blackman response that the slope is constrained to be zero at streamline lengths beyond that of the asymptote. To accommodate the possibility that the slope obtained empirically differs from zero, a piecewise linear model can therefore also be applied. The modelling steps described were undertaken in R (R Core Team 2019), through quantile regression, using the rq() and nlrq() functions provided in the “quantreg” package (Version 5.51, Koenker et al. 2018). The SSlinp() function in the “nlraa” package (Version 0.89, Miguez et al. 2021) was used as a self-starter for the coefficients of the Blackman function. Piecewise linear models were fitted using the “segmented” package (Version 1.3–4, Muggeo 2021). Mathematical descriptions of the functions are provided in the Supporting Information.

**Fig. 3:**
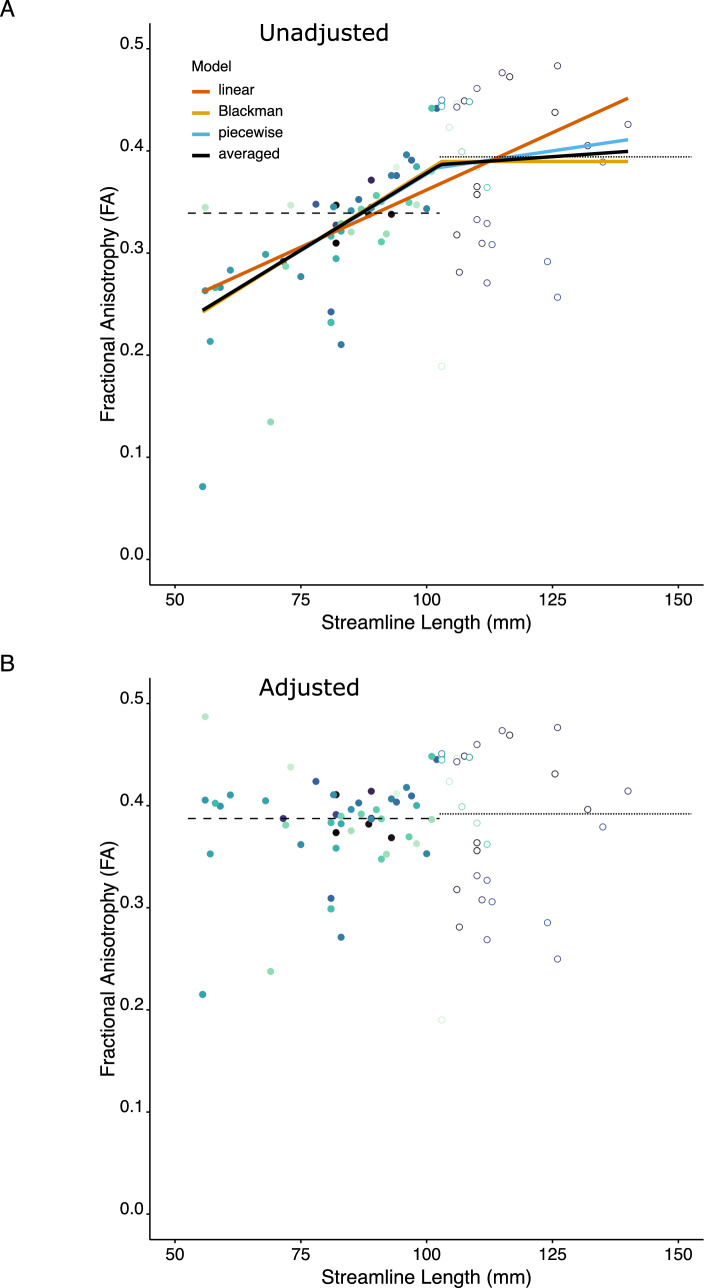
**A** Empirical fits to each of the three candidate models (linear (red), Blackman (orange), and piecewise-linear (blue), and the model averaged fit (black), are shown for the 84 tracts delineated for a single individual. The Akaike weights for the respective models were as follows: linear – 0.007; Blackman – 0.564; piecewise-linear – 0.270. The point of inflection was determined to be 103.0 mm for the Blackman model (FA = 0.390), 102.0 mm for the piecewise-linear model (FA = 0.383), and 102.7 mm for the averaged model (FA = 0.388). The slope of the initial segment was estimated to be 0.003 for the Blackman model, the piecewise-linear model, and the averaged model. The slope of the second segment was estimated to be 0.0002 for the averaged model. **B** The FA values, adjusted for the influence of streamline length (through the application of a model averaging approach), are plotted for the same individual. In both panels, the colours are assigned to tracts in the order in which they are listed in the source data. Values corresponding to streamline lengths shorter than the inflection point are assigned closed symbols, and values corresponding to streamline lengths longer than the inflection point are assigned open symbols. In both panels, the horizontal dotted line corresponds to the median FA value of tracts with streamline lengths longer than the inflection point. In each case, the horizontal dashed line corresponds to the median FA value of tracts with streamline lengths shorter than the inflection point

One of our goals was to derive a means of compensating for the association between streamline length and FA (and other quantitative estimates derived from diffusion tractography). To this end, we employed model-averaging. It has been demonstrated that model-averaged estimators improve precision and reduce bias, relative to estimators derived from a selected “best model” (Burnham and Anderson 2002). When the aim is prediction and the calculation of residuals – which is central to the present undertaking, model averaging is always superior to a best-model strategy (Burnham and Anderson 2004). Recognising that any model is merely an approximation to the truth, Akaike’s information criterion (AIC) was used to quantify the three candidate models (linear, Blackman, and piecewise-linear) in terms of information loss. In practice, the modified version (AICc), that is suitable for small sample sizes, was used. An Akaike weight was then calculated for each model (i.e., separately for each data sample/participant). The Akaike weight takes a value between 0 and 1. Importantly, the weights of all models in a candidate set sum to 1. The Akaike weight is analogous to the probability that any given model is the best approximating model of the data (Symonds and Moussalli 2011). A weighted average of model predictions is obtained by multiplying the predicted values generated by each model by the corresponding Akaike weight, and then summing across the (in this case three) sets of weighted predicted values (Burnham and Anderson 2002; Symonds and Moussalli 2011). A model with a low Akaike weight thus has little influence on prediction (Fig. 3A).

The model-averaging approach can also be applied to the individual parameter estimates generated by candidate models. The Blackman and the piecewise-linear models yield estimates of the inflection point (or “breakpoint”) that marks the termination of the initial segment. In addition, both models provide estimates of the slope of the initial segment, and of the slope of the second segment (which for the Blackman model is equal to zero). Model averaged estimates for these parameters were obtained in the manner described above (the Akaike weights were rescaled such that with the exclusion of the linear model the sum remained equal to 1).

**Fig. 4:**
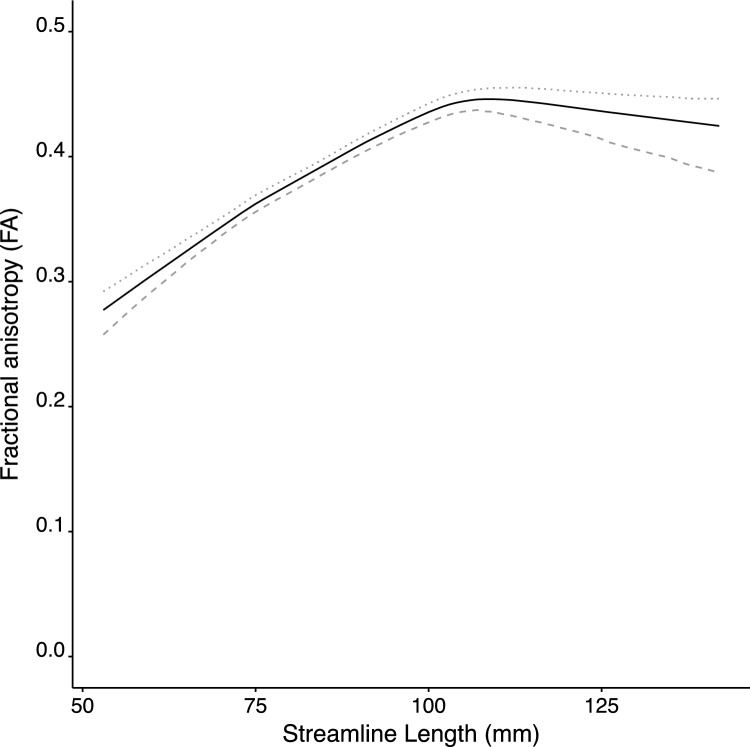
A summary representation of the results obtained through the application of a model averaging approach to the predicted FA values generated by the three candidate models (linear, Blackman, and piecewise-linear), for the 43 participants included in Ruddy et al. (2017). For each participant, the models were evaluated, and the predictions weighted and averaged, at a range of nominal streamline lengths (at 1 mm intervals). This range spanned the median minimum streamline length and the median maximum streamline length observed across the 43 participants. The solid line corresponds to the means of the weighted, averaged, predicted values derived from 1000 bootstrapped samples. The dashed line was generated using the lower 95% confidence interval of the bootstrapped samples. The dotted line was generated using the upper 95% confidence interval. It is apparent that, for streamlines shorter than approximately 100 mm, there is an association between FA and streamline length

The mean value of the inflection point (i.e., the transition between the two segments for the Blackman and piecewise models) calculated across participants was 102.1 mm (95% c.i. 98.6–104.8 mm). The confidence interval (c.i. = 6.2 mm) obtained for the inflection point was considerably smaller than that of the range of streamline lengths (c.i. = 26.0 mm) observed across participants. The mean value of the FA obtained at the inflection point was 0.45 (95% c.i. 0.44–0.46). The slope of the first segment was 0.0041 (95% c.i. 0.0034–0.0050). In other words, each 10 mm increase in streamline length was associated with an increase in FA of approximately 0.04. This indicates that over the range of streamline values spanned by the initial segment (i.e., up to approximately 102 mm), the FA estimate covaries with the length of the streamline for which it was derived. Consistent with this supposition, the Kendall tau values of correlations between streamline length and FA, calculated for streamlines shorter than that of the inflection point (derived separately for each individual) corresponded to large effect sizes (mean = 0.51, 95% c.i. 0.44–0.55). It is very clearly the case therefore that below the inflection point, FA estimates are in linear association with streamline length (Fig. 4).

In contrast, the mean slope of the second segment (Fig. 4) did not differ reliably from zero (mean =  – 0.0006, 95% c.i.  – 0.0014 to 0.00008). In this case, a 10 mm increase in streamline length was associated with a decrease in FA of 0.006. The correlation between streamline length and FA in this region was similarly weak (mean =  – 0.09, 95% c.i.  – 0.15 to  – 0.013).

The model-averaged (predicted) values represent the optimal fit to the relationship between streamline length and the quantitative metric (in this case FA), at the values of streamline length present in the data available for each individual participant. The residuals (Fig. 3B) correspond to the variations in the quantitative metric that are not predicted by streamline length. A positive value of the residual indicates that, for a given tract, the estimate is larger than that which would be predicted by streamline length alone – to an extent corresponding to its magnitude. A negative residual value for a tract indicates an estimate (e.g., of FA) smaller than would be predicted for its streamline length. The mean magnitude of the Kendall correlation between the residuals and streamline length, when calculated across participants, was  – 0.009 95% (c.i.  – 0.022 to 0.005). Calculated across tracts also, the correlation between the residuals and streamline length did not differ reliably from zero (mean =  – 0.003, 95% c.i.  – 0.054 to 0.045). The model averaging approach was therefore successful in compensating for the association between streamline length and FA.

The use of the residuals in inferential analyses may prove to be sufficient in circumstances in which the research question can be addressed by dealing with variations in the *relative magnitude* of the quantitative metric obtained for a defined set of tracts within each brain. There are however many instances in which it is desirable to compare the estimates (e.g., of FA) obtained for a specific tract, across groups of individuals, or to examine changes that occur in individuals over time. To address this requirement, it is necessary that the residuals are expressed relative to an appropriate reference value. The model-averaging approach affords a means of addressing this requirement. Specifically, the model averaged estimate of FA (or AFD etc.) at the inflection point provides an appropriate, individual specific, reference value. The magnitudes of the estimates obtained in the present analysis (median of 0.45 for FA across all individuals) have face validity. In so much as the fitted values of the model do not tend to vary appreciably over the range of streamline lengths extending beyond the inflection point (as indicated by the negligible slopes of the second segment), the model averaged estimate at the inflection point can reasonably be taken as an asymptotic value. Thus, it also has construct validity. It is therefore proposed that the adjusted (for streamline length) measure be obtained (i.e., separately for each brain) as the sum of the model averaged estimate of FA (or AFD etc.) at the inflection point, and the residual derived for each tract.

**Fig. 5:**
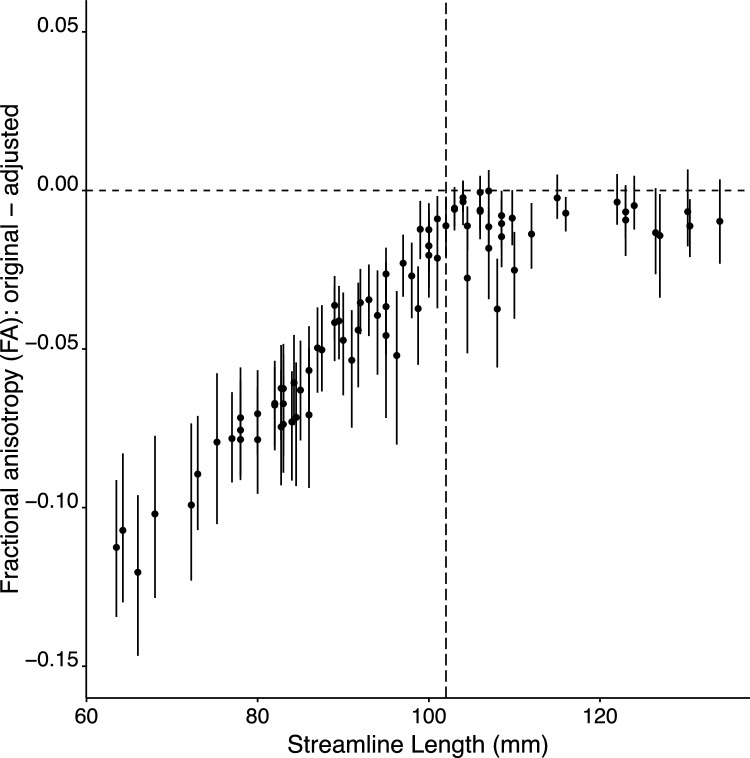
Separately for each of the 43 participants included in Ruddy et al. (2017), and for each tract, the difference between the original FA value, and the FA value adjusted for the influence of streamline length (through the application of a model averaging approach) was calculated. The filled symbols correspond to the means of these difference values, when derived from 1000 bootstrapped samples drawn from the set of 43 participants (i.e., calculated separately for each tract). For a tract to be included, it was necessary that at least half of the participants must have contributed data (i.e., streamlines were resolved). The error bars correspond to the 95% confidence intervals of the bootstrapped samples. The tracts are plotted in order of mean streamline length (calculated across participants)

The adjusted values derived in the manner described, differ systematically from the original FA values that are analysed routinely in tractography studies. Necessarily the magnitude of the difference varies as a function of streamline length (Fig. 5). Estimates generated for tracts with the shortest streamlines (typically < 70 mm) are characterised by adjusted FA values that exceed the original values by more than 0.1. In respect of tracts with streamline lengths greater than the mean inflection point of 102 mm (estimated across participants), the magnitude of the difference tends to be smaller, and frequently cannot be distinguished reliably from zero (Fig. 5).

**Fig. 6:**
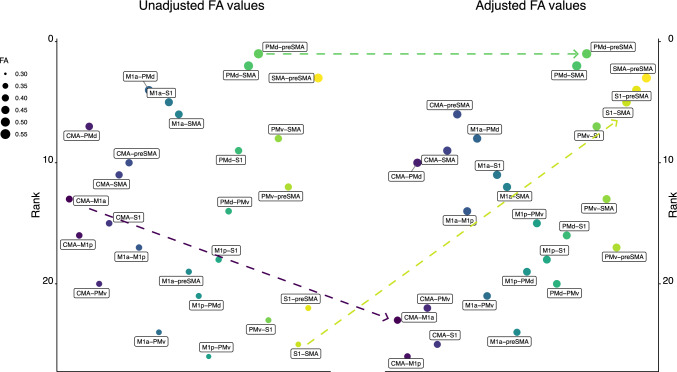
The left portion of the figure (“Unadjusted FA values”) displays the rank ordering of unadjusted FA values obtained for streamlines (n = 26 tracts) that originate and terminate within the right cerebral hemisphere. The right portion of the figure (“Adjusted FA values”) displays the rank ordering of FA values that have been adjusted for streamline length in the manner described in the text. The size of each symbol (in the legend shown in the range 0.30–0.55) corresponds to the associated FA value (i.e., derived for that tract). The position of each symbol in relation to the y axis scale corresponds to the ranking of the FA value with respect to the set of 26 tracts. Those with a higher ranking (larger FA value) appear above those with a lower ranking (smaller FA value). While the assignation of fill colour to the individual tracts is arbitrary, as it remains consistent across panels A and B, it aids in the identification of differences in ranking (i.e., unadjusted versus adjusted). In both panels, the colours (which do not relate to FA value) are assigned to tracts in the order in which they are listed in the source data. The x axis is categorical. Tracts are plotted with respect to the x axis in the order in which they are listed in the source data. It is apparent that the rank ordering of the adjusted values (Panel B) is dramatically different from that of the unadjusted values (Panel A). *M1a* anterior primary motor cortex, *M1p *posterior and primary motor cortex, *PMd* dorsal premotor cortex, *PMv* and ventral premotor cortex, *SMA* proper—supplementary motor area proper, *pre-SMA* pre-supplementary motor area, *S1- primary* sensory cortex, *CMA* cingulate motor area. Three exemplars are highlighted via arrows between the unadjusted and adjusted values. The adjusted ranking (and FA value) for the S1-SMA tract is markedly higher than the unadjusted ranking (and value). The adjusted ranking for the CMA-M1a tract is lower than the unadjusted ranking. In respect of the PMd-preSMA tract, the ranking of the adjusted and unadjusted FA values is the same

The broader implications of the distortions in FA values that arise from streamline length dependence are readily demonstrated. The panel on the left of Fig. 6 illustrates the rank ordering of unadjusted FA values obtained for streamlines (n = 26 tracts) that originate and terminate within the right cerebral hemisphere. The panel on the right of Fig. 6 illustrates the rank ordering of FA values that have been adjusted for streamline length in the manner described above. It is apparent that the rank ordering of the adjusted values is dramatically different from that of the unadjusted values. This is reflected in a correlation of the rankings for the unadjusted and unadjusted FA values that is markedly lower than unity (Kendall’s tau = 0.34).

**Fig. 7:**
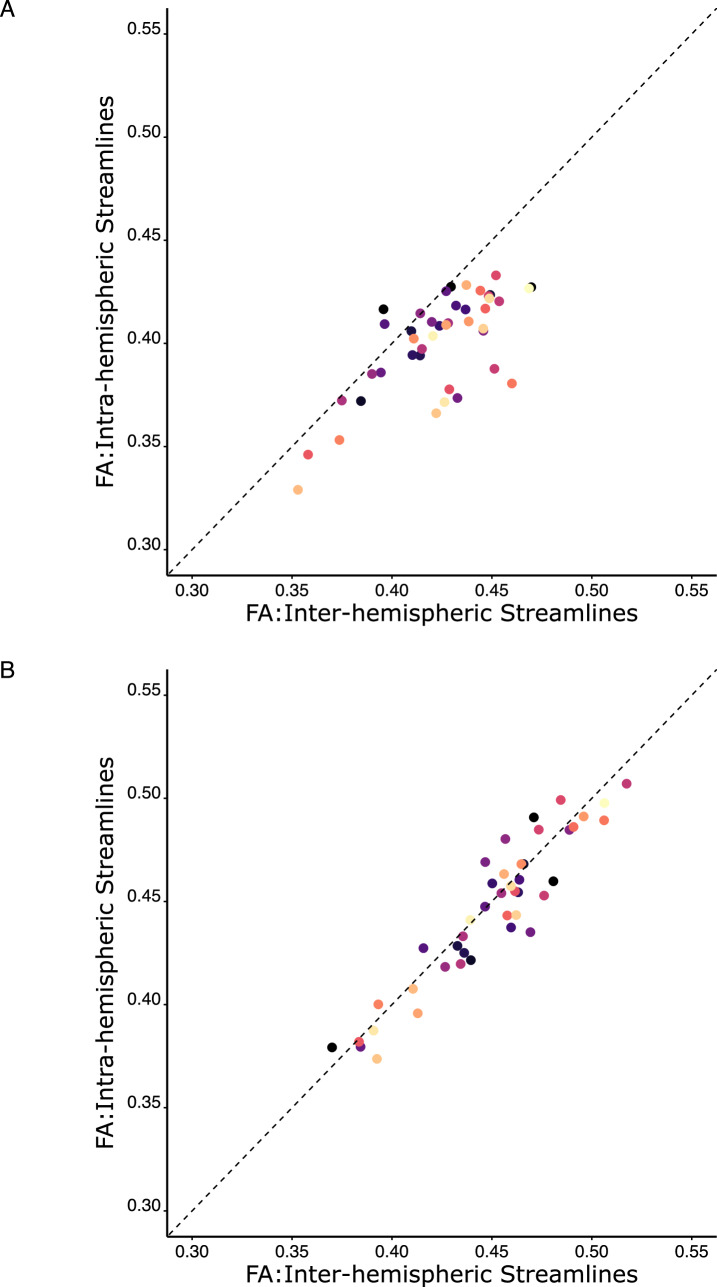
The trimmed mean (trimming = 20%) of the FA values derived for all inter-hemispheric tracts for which streamlines were resolved for each participant, and the trimmed mean of the FA values derived for all right hemisphere intra-hemispheric tracts for which streamlines were resolved for each participant, were calculated. Unadjusted FA values are shown in Panel **A**. FA values that have been adjusted for streamline length in the manner described in the text are shown in Panel **B**. Each point represents the data for a single participant, with the colour coding determined by the order in which the 43 participants are listed in the source data. In both panels, the x coordinate for each point corresponds to the trimmed mean FA value for the right hemisphere tracts, and the y coordinate corresponds to the trimmed mean FA value for the inter-hemispheric tracts. Points lying close to the line of equality indicate similarity in the FA values obtained for inter-hemispheric and right hemisphere streamlines. Points lying below the line of equality indicate FA values for the right hemisphere streamlines that are lower than those for the inter-hemispheric streamlines. Comparison of the plots generated for the unadjusted data (Panel **A**) and the adjusted data (Panel **B**) makes apparent that differences between right hemisphere and inter-hemispheric FA values present for the unadjusted data, are not apparent for the adjusted data. The results of corresponding inferential tests are reported in the text

It is not difficult to envisage specific research questions for which the outcomes may depend on whether the data have first been adjusted to compensate for the association with streamline length. For example, an investigator may wish to determine whether tracts that originate and terminate within one hemisphere differ from “inter-hemispheric” tracts, with respect to a metric such as FA. For the data set described, the central tendency (derived using trimmed means) of the unadjusted FA values (Fig. 7, Panel A) obtained for “intra-hemispheric” tracts within the right hemisphere is 0.404, whereas, for “inter-hemispheric” tracts connecting the left and right hemispheres, the corresponding value is 0.427 (difference =  – 0.022, 95% c.i.  – 0.031 to  – 0.015). The outcomes of a robust inferential test (Yuen’s t) appropriate for paired observations (i.e., intra-hemispheric versus inter-hemispheric) indicate that this is a large (in terms of standardised effect size) and reliable effect (t(26) =  – 5.17, p = 2.13E-05, δt =  – 0.59, 95% c.i.  – 0.81 to  – 0.39). In marked contrast, the difference between the adjusted FA values (Fig. 7, Panel B) obtained for the intra-hemispheric (0.448) and inter-hemispheric (0.453) tracts is – 0.004 (95% c.i.  – 0.009 – 0.0008). The associated inferential test (t(26) =  – 1.29, p = 0.175, δt =  – 0.08, 95% c.i.  – 0.22 to 0.01) supports the conclusion that, when adjusted for streamline length, the FA values obtained for streamlines defined for intra-hemispheric tracts do not differ reliably from those defined for inter-hemispheric tracts.

The model-averaging approach described is based on determining the optimal fit to the relationship between streamline length and the quantitative metric, at the values of streamline length present in the data available for each person. It is also possible to model the relationship between streamline length and a quantitative metric (e.g., FA), using the data available for each tract. In many instances however, this approach will be insufficient to provide an adequate characterisation of the relationship. This is because the extent of the variation across individuals in the length of the streamlines identified for a specific tract, will typically be much smaller than the range of the streamline lengths that characterise the entire set of tracts defined for each person. For the data considered herein, the mean (i.e., across 43 individuals) of the range of streamline lengths detected for all tracts (defined using the cortical motor network atlas) was 106.4 mm. In contrast, the mean for all tracts, of the range of streamline lengths obtained for each tract, was 63.9 mm. Necessarily also, the range of streamline lengths obtained (across persons) for a given tract will span only a subset of the range of streamline lengths defined for any given person.

**Fig. 8:**
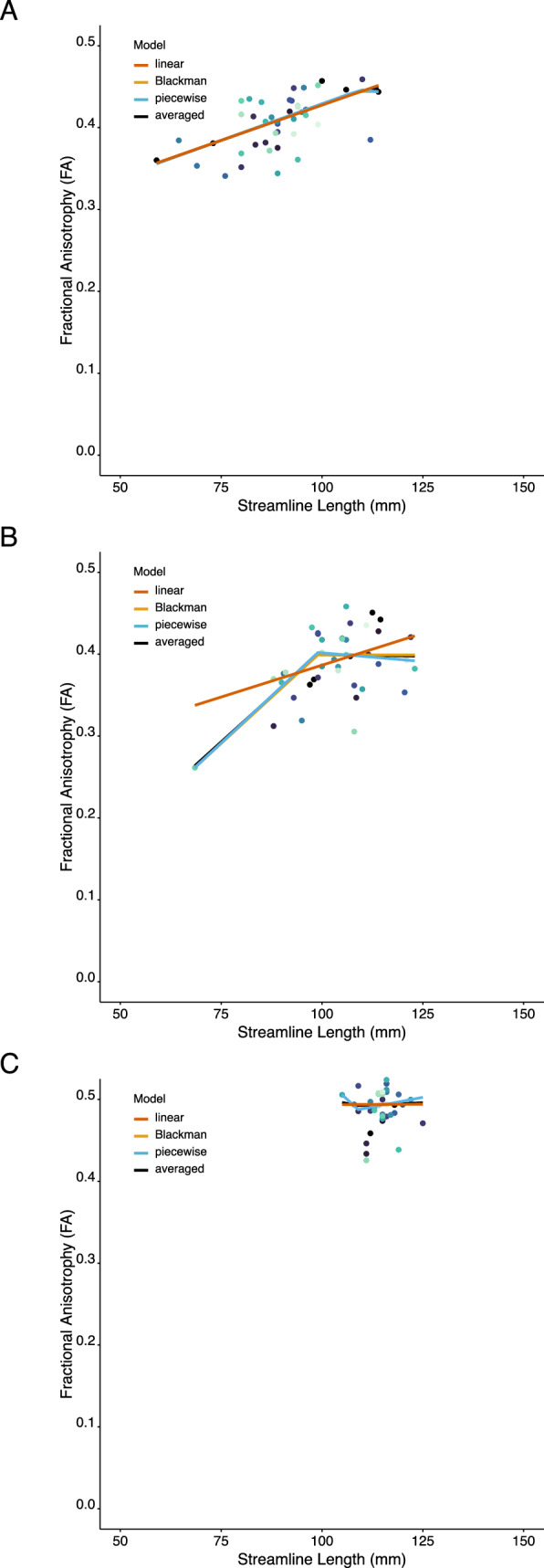
Empirical fits to each of the three candidate models (linear (red), Blackman (orange), and piecewise-linear (blue), and the model averaged fit (black), are shown for three example tracts. Each point represents the data for a single participant, with the colour coding determined by the order in which the 43 participants are listed in the source data. **A**. Between left cingulate motor area (CMA) and left dorsal premotor cortex (PMd). **B** Between left CMA and right anterior primary motor cortex (M1a). **C** Between left PMd and right PMd

The impact of these particulars on the data modelling is illustrated by Fig. 8. In Panel A, the left cingulate motor area to left dorsal premotor cortex tract (PMd) is represented. For 39 of the 43 individuals, the length of the streamlines defined for this tract was shorter than the point of inflection defined for the FA values (102.1 mm). For this range of streamline lengths, the relationship with FA is predominantly linear. In Panel B, the left CMA to right anterior primary motor cortex (M1a) tract is represented. For 18 of the 42 individuals for whom streamlines were detected, these were shorter than the FA point of inflection. Whereas, for 24 individuals, the streamlines were longer than the inflection point. In this case, a piecewise linear relationship is apparent. In Panel C, the left PMd to right PMd tract is shown. For all 43 individuals, the lengths of the streamlines detected for this tract exceeded the point of inflection defined for FA. With respect to this tract, there is no apparent relationship between streamline length and FA. These examples serve to highlight that, an adequate characterisation of the relationship that exists between streamline length and this tractography-derived metric (i.e., FA), demands a range of streamline lengths beyond that which is available for a single tract.

## Discussion

We have demonstrated that there is a clear and robust association between FA and the length of the streamlines for which this quantitative metric is derived. The relationship is described well as piecewise linear (Fig. 4). In our illustration, we showed that for a range of streamline lengths below a point of inflection, each 10 mm increase in streamline length was associated with an increase in FA of approximately 0.04 In other words, if two tracts both with streamlines shorter than 100 mm or so, vary in their respective streamline lengths by 25 mm, there will tend to be a difference of approximately 0.1 in the magnitude of their FA estimates. For streamlines longer than the point of inflection, the association is very much weaker, with the slope of the relationship between streamline length and FA not differentiable from zero.

It was illustrated (Fig. 1) that an influence of streamline length on an idealised quantitative metric can arise if the values (i.e., corresponding to FA or another quantitative metric) diminish progressively towards the ends of a streamline (Zhang et al. 2018), and a fixed threshold is used (i.e., by a tractography algorithm) to terminate tracking. The specific relationship between streamline length and FA observed empirically (Fig. 4) arises from the fact that in real brains, the estimate derived for any given specimen will be less than the theoretical upper limit defined for that measure (Clark et al. 2001) (Fig. 1, Panel V). As the observed relationship appears therefore to have a principled basis, it seems probable that it will be ubiquitous (see Supplementary Information). Given that the dependency is most pronounced for a range of streamline lengths encountered typically in (human brain) DW imaging (i.e., shorter than 100 mm or so), many previous estimates of tractography-derived metrics such as FA should be viewed with extreme caution. Indeed, it is only tracks composed of streamlines with lengths that exceed the inflection point, for which the estimates are likely to be veridical.

Due to the piecewise linear nature of the relationship between streamline length and FA (and other related metrics), it will not generally be feasible to adjust for its influence by simply including streamline length as a linear covariate in downstream analyses (e.g., Vos et al. 2011). There may be exceptions, if for example it can be established that the range of streamline lengths for all tracts is below the inflection point defined for the specimen in question. There is however a more secure alternative. We have outlined that an Akaike information weighted average of linear, Blackman and piecewise linear model predictions, may be used to compensate effectively for the dependence of FA on streamline length – across the entire range of streamline lengths present in each specimen. If the research question requires only consideration of the relative magnitude (within each brain) of the estimates (e.g., of FA) obtained for a specific set of tracts, it may be sufficient to employ the residuals of the weighted average model predictions. In many cases however, it will be desirable to compare the values obtained for a specific tract across different samples. We have proposed that the estimated value obtained at the inflection point of the weighted average model, provides a basis upon which to adjust the residuals derived for each brain, in a manner sufficient to permit valid comparisons across samples.

These considerations emphasise the importance of including as many tracts as possible in the modelling process, to best characterise the nature of the streamline length association. It is particularly desirable to include tracts with lengths sufficient to define the inflection point for each specimen (i.e., each participant). In practice therefore, it may be necessary to incorporate in the model, tracts that are not of direct interest in the analyses that follow. We have shown that the strategy of determining the optimal fit to the relationship between streamline length and a quantitative metric (e.g., FA), at the values of streamline length present in the data available for each person, is preferable to modelling the relationship between streamline length and that metric using data available for each tract (i.e., across persons). This is since the extent of the variation across individuals in the length of the streamlines identified for a given tract, will usually be much smaller than the range of the streamline lengths that characterise the entire set of tracts defined for each person. It remains to be determined whether potential inhomogeneities in dispersion patterns across brain regions call for statistical models that include subsets of tracts defined using spatio-anatomical criteria. As our recommendation is for the modelling to be conducted at the level of the individual (i.e., using the data derived for a single brain), the size of the cohort from which that individual is drawn will have little bearing on the goodness of fit or adequacy of compensation.

The representations shown in Fig. 1 are based upon certain assumptions derived from empirical data (e.g., estimates of FA diminish progressively towards the ends of a streamline) and reflect details of algorithms applied in tractography (such as magnitude thresholding). While this Gedankenexperiment influenced the choice of functions that were applied to the sample data, it is however critical to emphasise that the associations revealed by our analyses were derived through application of a statistical model, rather than a biophysical model. In this vein also, compensation for the associations was achieved using the same statistical methods. We have shown (in the Supporting Information) that these functions can be applied to characterise (and compensate for) associations between streamline length and several other quantitative metrics commonly derived from diffusion tractography. The reader might reasonably enquire whether the associations generalise, for example, to different age groups. In this regard, the primary data were for a pool of participants with a mean age of 22.5 years. The individuals included in Data set 2 (Supporting Information), for whom the same associations were present, had a mean age of 44.2 years. Although the matter must be resolved empirically (i.e., for other age groups), our presumption is that the associations are not age dependent.

A further question concerns the degree to which the reported effects are specific only for tractography but not, for example, voxel-based tract analysis. The statistical method described here is most obviously applicable to instances in which measures of central tendency (for some diffusion metric) are generated for an entire tract (and the relative influence of “endpoint effects” varies in accordance with streamline length). In the case of voxel-based tract analysis, in principle, similar associations between tract length and the derived quantitative metric may arise due to spatial smoothing (Jones et al. 2005), as this increases “effective partial voluming” (Smith et al. 2006). Partial volume effects (PVE) are attributable to the inclusion within a voxel of some non-fibre partial volume fraction. It has been shown previously that PVE modulate the values of diffusion metrics such as FA and MD (Vos et al. 2011). Necessarily such effects are most prominent for the terminal parts of white matter tracts. Indeed, it has been proposed that signal fraction maps be used to terminate fibre tracking (Guo et al. 2020). Thus, to the extent that spatial smoothing, is applied in voxel-based tract analysis, associations between the defined fibre length and diffusion metrics (reflecting contributions of PVE that are proportionally greater for shorter fibres) may be anticipated. Since estimates of the orientation dispersion particularly and, to a lesser extent, the intra-cellular volume fraction, are related to FA (Zhang et al. 2012), similar effects may emerge when smoothing (e.g., Lehmann et al. 2021) is applied to Neurite Orientation Dispersion and Density Imaging (NODDI) modelled data. It is known that quantitative metrics such as FA covary with streamline count (Correia et al. 2008). The number of streamlines retained for quantitative analysis can be influenced by the application of various filtering schemes (e.g., SIFT, COMMIT, LiFE). Although none of these schemes provide more than a modest improvement in connectome accuracy (with respect to “ground truth”), they appear to be most effective (in retaining true positives and rejecting false positives) for short-range connections (1–25 mm). For longer connections (26–160 mm) the removal of false positives via filtering may be less successful (Sarwar et al. 2023). Although the use of such filters may therefore disproportionately increase the number of medium to long range streamlines that are retained, it is challenging to predict the magnitude of any consequential effect on the relationships described herein. A more general point is that the presence or extent of an association between a specific quantitative diffusion metric and streamline or tract length, whether this be in the context of a different imaging protocol (e.g., single shell versus multi-shell) or novel signal decomposition technique (e.g., Huynh et al. 2020) cannot always be determined a priori. Statistical modelling is likely to be required. In a similar vein, the statistical functions necessary to adequately model a particular association may differ from those which have been applied in the present case.

The impact of the associations characterised in the manuscript may not extend in a straightforward fashion to analytic approaches in which quantitative estimates (e.g., of FA) are derived at specific positions along a “Tract Profile” (Yeatman et al. 2012), rather than as measures of central tendency calculated for an entire tract. It is important to note however, that in the method described by Yeatman et al. (2012) only the central portion of each streamline is used to generate quantitative metrics, as this is deemed to be “generally consistent across individuals” (page 13). In the case of shorter tracts however (see our Fig. 1), PVE may still exert a disproportionate influence on the “central portion”, which is defined by Yeatman et al. (2012) on statistical grounds (e.g., less than 4 standard deviations above the mean fiber length). In the approach presented herein, the influence of the non-central portion on quantitative metrics is modelled explicitly. It remains to be determined whether this strategy has the potential to yield reliable information concerning individual differences beyond that which can be realised by restricting analysis to the central portion only.

## Supplementary Information

Below is the link to the electronic supplementary material.

Supplementary file1 (DOCX 363 KB)

## Data Availability

Example R code, which can be used to implement the methods of analysis described herein, is available via: https://zenodo.org. The Digital Object Identifier (DOI) is: 10.5281/zenodo.10808421. In respect of the brain imaging files upon which these analyses are based, three participants provided consent for their exemplar, pseudo-anonymised, data to be placed in the public domain. The example R code can be used to analyse these data, which are made available at the same location.
