# Supplementary material for: Quantitative metrics commonly derived from diffusion tractography covary with streamline length: a characterization and method of adjustment
Source: Brain Struct Funct. 2024 Sep 11;229(9):2431–44. doi: 10.1007/s00429-024-02854-9 (PMC11611944; doi:10.1007/s00429-024-02854-9)
Supplement: Supplementary file 1 — Supplementary file1 (DOCX 363 KB) [file 429_2024_2854_MOESM1_ESM.docx]

**Quantitative metrics commonly derived from diffusion tractography covary with streamline length: A characterization and method of adjustment.**

**Brain Structure and Function**

Richard G. Carson^a,b^ and Alexander Leemans^c^

^a^Trinity College Institute of Neuroscience and School of Psychology, Trinity College Dublin, Dublin 2, Ireland

^b^School of Psychology, Queen's University Belfast, Belfast, Northern Ireland, BT7 1NN, UK.

^c^Image Sciences Institute, University Medical Center Utrecht, Utrecht 85500, The Netherlands.

Corresponding Author:

E-mail: richard.carson@tcd.ie

**Supporting Information**

Mathematical description of the functions

where

y represents the value of the quantitative metric (e.g., FA),

x is the streamline length (mm),

a is the intercept (the value of y when streamline length = 0),

b_i_ is the slope of the initial segment (as the change in y per unit of streamline length),

b_s_ is the slope of the second segment (as the change in y per unit of streamline length),

j is the inflection point (mm) – at which the second segment starts,

z is the value of the quantitative metric at the point of inflection (mm).

*Linear*

y = 𝑎+ b_i_⋅𝑥

*Blackman*

$$\left\{ \begin{aligned} x < j, y = a + b_{i}\cdot x \\ x \geq j, y = a + b_{i}\cdot j \end{aligned} \right.$$

*Piecewise linear*

$$\left\{ \begin{aligned} x < j, y = a + b_{i}\cdot x \\ x \geq j, y = z + b_{s}\cdot(x-j) \end{aligned} \right.$$

Data Set 1

Further analyses using the methods detailed in the paper, were applied to the data described in Ruddy et al. (2017). These demonstrated that quantitative metrics derived from diffusion tractography other than fractional anisotropy (FA) also covary with streamline length. While the analyses corroborate the supposition that the associations are materially grounded, it is also important to note that several commonly reported quantitative indices are mutually interdependent. For example, specific values of FA (and mean diffusivity (MD)) may arise from different combinations of radial diffusivity (RD) and axial diffusivity (Alexander et al., 2007). Similarly, estimates of apparent fibre density (AFD) vary with changes in RD (Raffelt et al., 2012).

As an initial step, the Kendall rank correlation coefficient was used to characterise the ordinal association of streamline length and, respectively, AFD, MD and RD. This was done in two ways. In the first, the coefficient was calculated separately for each of the 43 participants. The mean value of tau – the Kendall correlation coefficient, was then obtained (with corresponding confidence intervals) across participants (Table S1). Within individual brains, tracts with longer streamlines were characterised by larger AFD and MD values, and by smaller RD values.

**Table S1**

|  | lower c.i. | mean | upper c.i. |
| --- | --- | --- | --- |
| AFD vs. streamline length | 0.33 | 0.38 | 0.42 |
| MD vs. streamline length | 0.05 | 0.11 | 0.16 |
| RD vs. streamline length | -0.30 | -0.26 | -0.22 |

Kendall correlation coefficients (tau) which characterise the magnitude of the association between each quantitative estimate and streamline length - when the coefficients were calculated separately for each of the 43 participants. The means and confidence intervals (c.i.) have been calculated over all (n = 43) participants.

In the second method of analysis, the coefficient was calculated separately for each of the tracts (e.g., left M1a to right M1a), using the sample of 43 participants. The mean value of tau across all tracts obtained (with corresponding confidence intervals) was then derived (Table S2). It is apparent that when any given tract is considered across different brains, individuals with longer streamlines tend to exhibit larger AFD and MD values, and smaller RD values.

**Table S2**

|  | lower c.i. | mean | upper c.i. |
| --- | --- | --- | --- |
| AFD vs. streamline length | 0.25 | 0.29 | 0.32 |
| MD vs. streamline length | 0.02 | 0.05 | 0.08 |
| RD vs. streamline length | -0.22 | -0.18 | -0.14 |

Kendall correlation coefficients (tau) which characterise the magnitude of the association between each quantitative estimate and streamline length - when the coefficients were calculated separately for each tract. The means and confidence intervals (c.i.) have been calculated over all (n = 43) participants.

Further analyses were then undertaken to determine for each quantitative estimate, the nature of the relationship with streamline length. Model averaged estimates (with confidence intervals) derived using linear, Blackman and piecewise linear models were obtained in the manner described in the paper.

Apparent fibre density (AFD)

In 26 of the 43 participants, the fit to the piecewise linear function was superior to that achieved using a Blackman (n = 15) or linear model (n = 2). The model averaged estimates (with 95% confidence intervals (c.i.)) are given in Table S3.

**Table S3**

|  | lower c.i. | estimate | upper c.i. |
| --- | --- | --- | --- |
| breakpoint (mm) | 99.4 | 103.2 | 107.2 |
| AFD at breakpoint | 0.76 | 0.79 | 0.82 |
| slope initial segment | 0.0075 | 0.0084 | 0.0093 |
| slope second segment | -0.0049 | -0.0026 | -0.0012 |
| tau pre-breakpoint | 0.48 | 0.52 | 0.55 |
| tau post-breakpoint | -0.16 | -0.09 | -0.03 |

A summary of the values obtained through the application of a model averaging approach to the parameter estimates generated by the three candidate models (linear, Blackman, and piecewise-linear). The lower and upper 95% confidence intervals were generated from 1000 bootstrapped samples (drawn from the cohort of forty-three participants). The slope values represent the change in AFD with respect to each 1 mm increase in streamline length, for i) the initial segment, ii) the second segment. The tau values are the Kendall correlation coefficients for these segments. These characterise the magnitude of the association between AFD and streamline length.

The mean magnitude of the Kendall correlation between AFD values adjusted for the influence of streamline length (i.e., through the application of a model averaging approach) and streamline length, when calculated across participants, was -0.021 (95% c.i. -0.038 – -0.006). Calculated across tracts, the correlation between the (streamline length) adjusted AFD and streamline length did not differ reliably from zero (mean = -0.023, 95% c.i. -0.067 – 0.020).

Figure S1. A summary representation of the results obtained through the application of a model averaging approach to the predicted AFD values generated by the three candidate models (linear, Blackman, and piecewise-linear), for the 43 participants included in Ruddy et al. (2017). For each participant, the models were evaluated, and the predictions weighted and averaged, at a range of nominal streamline lengths (at 1mm intervals). This range spanned the median minimum streamline length and the median maximum streamline length observed across the 43 participants. The solid line corresponds to the means of the weighted, averaged, predicted values derived from 1000 bootstrapped samples. The dashed line was generated using the lower 95% confidence interval of the bootstrapped samples. The dotted line was generated using the upper 95% confidence interval. It is apparent that, for streamlines shorter than approximately 100 mm, there is a clear association between AFD and streamline length.

Figure S2. Separately for each of the 43 participants included in Ruddy et al. (2017), and for each tract, the difference between the original AFD value, and the AFD value adjusted for the influence of streamline length (through the application of a model averaging approach) was calculated. The filled symbols correspond to the means of these difference values, when derived from 1000 bootstrapped samples drawn from the set of 43 participants (i.e., calculated separately for each tract). For a tract to be included, it was necessary that at least half of the participants must have contributed data (i.e., streamlines were resolved). The error bars correspond to the 95% confidence intervals of the bootstrapped samples. The tracts are plotted in order of mean streamline length (calculated across participants).

Mean diffusivity (MD)

In 28 of the 43 participants, the fit to the piecewise linear function was superior to that achieved using a Blackman (n = 3) or linear model (n = 12). The model averaged estimates (with 95% confidence intervals (c.i.)) are given in Table S4.

**Table S4**

|  | lower c.i. | estimate | upper c.i. |
| --- | --- | --- | --- |
| breakpoint (mm) | 88.1 | 105.4 | 123.6 |
| MD at breakpoint | 6.90E-04 | 6.96E-04 | 7.00E-04 |
| slope initial segment | -3.97E-06 | -1.67E-06 | -2.76E-07 |
| slope second segment | 2.08E-07 | 4.32E-07 | 7.77E-07 |
| tau pre-breakpoint | -0.01 | 0.07 | 0.14 |
| tau post-breakpoint | 0.05 | 0.12 | 0.18 |

A summary of the values obtained through the application of a model averaging approach to the parameter estimates generated by the three candidate models (linear, Blackman, and piecewise-linear). The lower and upper 95% confidence intervals were generated from 1000 bootstrapped samples (drawn from the cohort of forty-three participants). The slope values represent the change in MD with respect to each 1 mm increase in streamline length, for i) the initial segment, ii) the second segment. The tau values are the Kendall correlation coefficients for these segments. These characterise the magnitude of the association between MD and streamline length.

When calculated across participants, the mean magnitude of the Kendall correlation between the MD values adjusted for the influence of streamline length (i.e., through the application of a model averaging approach) and streamline length did not differ reliably from zero (mean = 0.002, 95% c.i. -0.015 – 0.017). The mean magnitude of the Kendall correlation between the (streamline length) adjusted MD values and streamline length, when calculated across tracts, was 0.045 (95% c.i. 0.018 – 0.068).

Figure S3. A summary representation of the results obtained through the application of a model averaging approach to the predicted MD values generated by the three candidate models (linear, Blackman, and piecewise-linear), for the 43 participants included in Ruddy et al. (2017). For each participant, the models were evaluated, and the predictions weighted and averaged, at a range of nominal streamline lengths (at 1mm intervals). This range spanned the median minimum streamline length and the median maximum streamline length observed across the 43 participants. The solid line corresponds to the means of the weighted, averaged, predicted values derived from 1000 bootstrapped samples. The dashed line was generated using the lower 95% confidence interval of the bootstrapped samples. The dotted line was generated using the upper 95% confidence interval.

Figure S4. Separately for each of the 43 participants included in Ruddy et al. (2017), and for each tract, the difference between the original MD value, and the MD value adjusted for the influence of streamline length (through the application of a model averaging approach) was calculated. The filled symbols correspond to the means of these difference values, when derived from 1000 bootstrapped samples drawn from the set of 43 participants (i.e., calculated separately for each tract). For a tract to be included, it was necessary that at least half of the participants must have contributed data (i.e., streamlines were resolved). The error bars correspond to the 95% confidence intervals of the bootstrapped samples. The tracts are plotted in order of mean streamline length (calculated across participants).

Radial diffusivity (RD)

In 29 of the 43 participants, the fit to the piecewise linear function was superior to that achieved using a Blackman (n = 8) or linear model (n = 6). The model averaged estimates (with 95% confidence intervals (c.i.)) are given in Table S5.

**Table S5**

|  | lower c.i. | estimate | upper c.i. |
| --- | --- | --- | --- |
| breakpoint (mm) | 99.2 | 103.6 | 107.4 |
| RD at breakpoint | 5.01E-04 | 5.08E-04 | 5.16E-04 |
| slope initial segment | -4.38E-06 | -2.68E-06 | -1.49E-06 |
| slope second segment | 4.83E-07 | 9.41E-07 | 1.49E-06 |
| tau pre-breakpoint | -0.46 | -0.42 | -0.36 |
| tau post-breakpoint | 0.05 | 0.15 | 0.22 |

A summary of the values obtained through the application of a model averaging approach to the parameter estimates generated by the three candidate models (linear, Blackman, and piecewise-linear). The lower and upper 95% confidence intervals were generated from 1000 bootstrapped samples (drawn from the cohort of forty-three participants). The slope values represent the change in RD with respect to each 1 mm increase in streamline length, for i) the initial segment, ii) the second segment. The tau values are the Kendall correlation coefficients for these segments. These characterise the magnitude of the association between RD and streamline length.

The mean magnitude of the Kendall correlation between RD values adjusted for the influence of streamline length (i.e., through the application of a model averaging approach) and streamline length, did not differ reliably from zero (mean = -0.010, 95% c.i. -0.029 – 0.009). Likewise, when calculated across tracts, the mean magnitude of the Kendall correlation between the (streamline length) adjusted RD values and streamline length did not differ reliably from zero (mean = 0.017, 95% c.i. -0.020 – 0.056)

Figure S5. A summary representation of the results obtained through the application of a model averaging approach to the predicted RD values generated by the three candidate models (linear, Blackman, and piecewise-linear), for the 43 participants included in Ruddy et al. (2017). For each participant, the models were evaluated, and the predictions weighted and averaged, at a range of nominal streamline lengths (at 1mm intervals). This range spanned the median minimum streamline length and the median maximum streamline length observed across the 43 participants. The solid line corresponds to the means of the weighted, averaged, predicted values derived from 1000 bootstrapped samples. The dashed line was generated using the lower 95% confidence interval of the bootstrapped samples. The dotted line was generated using the upper 95% confidence interval.

Figure S6. Separately for each of the 43 participants included in Ruddy et al. (2017), and for each tract, the difference between the original RD value, and the RD value adjusted for the influence of streamline length (through the application of a model averaging approach) was calculated. The filled symbols correspond to the means of these difference values, when derived from 1000 bootstrapped samples drawn from the set of 43 participants (i.e., calculated separately for each tract). For a tract to be included, it was necessary that at least half of the participants must have contributed data (i.e., streamlines were resolved). The error bars correspond to the 95% confidence intervals of the bootstrapped samples. The tracts are plotted in order of mean streamline length (calculated across participants).

Data Set 2

To demonstrate that the covariation of quantitative metrics derived from diffusion tractography with streamline length is observed more generally, the analysis of a different data set is reported. The data were obtained in the context of a study described in Hänggi et al. (2017) and Saettaet al. (2022). The participants were sixteen neurologically healthy males (aged 44.18 ± 9.51 SD). All gave informed consent to procedures that were in accordance with the Declaration of Helsinki. These had been approved by Ethics Committee of the University Hospital of Zurich and by the Ethical Committee Milano Area C. Eight participants were enrolled in Zurich, and eight in Milan.

At the University Hospital of Zurich, diffusion weighted imaging (DWI) was undertaken using a Philips Achieva 3.0 Tesla whole-body scanner (Philips Medical Systems, Best, The Netherlands) was used in conjunction with an eight-element sensitivity encoding (SENSE) head coil array. The DWI sequence was characterised by a repetition time of 13.010 s, echo time = 55 ms, b value = 1000 s/mm^2^, flip angle = 90°, spatial resolution = 2 × 2 × 2 mm^3^  (matrix 112 × 112 pixels, number of slices = 75 (transverse)), field of view = 224 × 224 mm^2^, SENSE factor, 2.1. Diffusion was registered in 32 noncollinear directions. A non-diffusion weighted reference volume (b = 0 s/ mm^2^) was also first obtained. At the Neuroradiology Department of the “ASST Grande Ospedale Metropolitano Niguarda" of Milan, the data were acquired using a General Electric (GE) 1.5 Tesla Signa HD-XT scanner. The DWI sequence was characterised by a b value of 700 s/mm^2^. Diffusion was registered in 35 noncollinear directions, with a spatial resolution of 1.094  × 1.094 × 4 mm^3^ . A preceding non-diffusion weighted reference volume (b = 0 s/ mm^2^) was obtained.

ExploreDTI (Leemans et al. 2009) was used for data processing. Images were corrected for head movement and eddy currents using the procedure described in Leemans and Jones (2009). Diffusion tensor estimation was performed using the iteratively reweighted linear least squares approach (Veraart et al. 2013). Whole-brain deterministic tractography was undertaken with a seed point resolution of 2 x 2 x 2 mm and a Fractional Anisotropy (FA) threshold of 0.2.

Reconstructed fibre trajectories were generated for all pairwise combinations of brain regions of interest (RoI) defined within the automated anatomical labelling (AAL) atlas (Rolls et al., 2015). This encompasses 90 RoIs for each hemisphere, including the whole cerebrum and subcortical structures, but excluding the cerebellum. In total, 4005 unique tracts (within and between hemispheres) are thus defined. The median number of tracts for which streamlines were present (i.e., across the sixteen participants) was 1342.2 (95% c.i. 1219.9 – 1461.2). For every such instance, estimates of fractional anisotropy (FA) and streamline length were derived. The relationship between FA and streamline length was analysed separately for each participant in the manner described in the main text.

In 11 of the 16 participants, the fit to the piecewise linear function was superior to that achieved using a Blackman model (n = 5). In no instance was the best fit achieved using a linear model. A summary of the fitted parameters obtained for the cohort of sixteen participants is provided in Table S6. The mean range of streamline lengths included in the analyses (i.e., across the sixteen participants) was 300.1 mm (95% c.i. 258.9 – 345.7 mm). A summary representation of the results obtained through the application of a model averaging approach to the predicted FA values generated by the three candidate models (linear, Blackman, and piecewise-linear) is shown in Figure S7.

**Table S6**

|  | lower c.i. | estimate | upper c.i. |
| --- | --- | --- | --- |
| breakpoint (mm) | 111.4 | 123.5 | 144.6 |
| FA at breakpoint | 0.46 | 0.47 | 0.48 |
| slope initial segment | 0.0013 | 0.0015 | 0.0017 |
| slope second segment | -1.87E-04 | -1.98E-05 | 1.22E-04 |
| tau pre-breakpoint | 0.35 | 0.37 | 0.40 |
| tau post-breakpoint | 0.03 | 0.12 | 0.15 |

A summary of the values obtained through the application of a model averaging approach to the parameter estimates generated by the three candidate models (linear, Blackman, and piecewise-linear). The lower and upper 95% confidence intervals were generated from 1000 bootstrapped samples (drawn from the cohort of sixteen participants). The slope values represent the change in FA with respect to each 1 mm increase in streamline length, for i) the initial segment, ii) the second segment. The tau values are the Kendall correlation coefficients for these segments. These characterise the magnitude of the association between FA and streamline length.

**Figure S7**

A summary representation of the results obtained through the application of a model averaging approach to the predicted FA values generated by the three candidate models (linear, Blackman, and piecewise-linear) for the 16 participants. For each participant, the models were evaluated, and the predictions weighted and averaged, at a range of nominal streamline lengths (at 1mm intervals). This range spanned the mean minimum streamline length (50 mm) and the mean maximum streamline length (346 mm) observed across the 16 participants. The solid line corresponds to the means of the weighted, averaged, predicted values derived from 1000 bootstrapped samples. The dashed line was generated using the lower 95% confidence interval of the bootstrapped samples. The dotted line was generated using the upper 95% confidence interval.

**References**

Alexander, A. L., Lee, J. E., Lazar, M., & Field, A. S. (2007). Diffusion tensor imaging of the brain. *Neurotherapeutics*, *4*(3), 316-329.

Hänggi, J., Vitacco, D. A., Hilti, L. M., Luechinger, R., Kraemer, B., & Brugger, P. (2017). Structural and functional hyperconnectivity within the sensorimotor system in xenomelia. *Brain and Behavior*, *7*(3), e00657.

Leemans, A., & Jones, D. K. (2009). The B‐matrix must be rotated when correcting for subject motion in DTI data. *Magnetic Resonance in Medicine: An Official Journal of the International Society for Magnetic Resonance in Medicine*, *61*(6), 1336-1349.

Leemans A, Jeurissen B, Sijbers J (2009) ExploreDTI: a graphical toolbox for processing, analyzing, and visualizing diffusion MR data. 17th Annual Meeting of Intl Soc Mag Reson Med, Hawaii, USA, p. 3537

Raffelt, D., Tournier, J.D., Rose, S., Ridgway, G.R., Henderson, R., Crozier, S., Salvado, O. & Connelly, A., (2012). Apparent fibre density: a novel measure for the analysis of diffusion-weighted magnetic resonance images. Neuroimage, 59(4), 3976-3994.

Rolls, E. T., Joliot, M., & Tzourio-Mazoyer, N. (2015). Implementation of a new parcellation of the orbitofrontal cortex in the automated anatomical labeling atlas. *Neuroimage*, *122*, 1-5.

Ruddy, K. L., Leemans, A., & Carson, R. G. (2017). Transcallosal connectivity of the human cortical motor network. *Brain Structure and Function*, *222*(3), 1243-1252.

Saetta, G., Ruddy, K., Zapparoli, L., Gandola, M., Salvato, G., Sberna, M., Bottini, G., Brugger, P. & Lenggenhager, B. (2022). White Matter Abnormalities in Body Integrity Dysphoria. Cortex.

Veraart, J., Sijbers, J., Sunaert, S., Leemans, A., & Jeurissen, B. (2013). Weighted linear least squares estimation of diffusion MRI parameters: strengths, limitations, and pitfalls. *Neuroimage*, *81*, 335-346.
